# Staging of the estrous cycle and induction of estrus in experimental rodents: an update

**DOI:** 10.1186/s40738-020-00074-3

**Published:** 2020-03-14

**Authors:** Ayodeji Folorunsho Ajayi, Roland Eghoghosoa Akhigbe

**Affiliations:** Department of Physiology, College of Medicinek, Ladoke Akintola University of Technology, Ogbomoso, Oyo, Nigeria

**Keywords:** Estradiol, Progesterone, Estrous cycle, Menstrual cycle, Reproductive cycle

## Abstract

**Background:**

Determination of the phases of the estrous cycle and induction of estrus (heat) in experimental animals remains useful, especially in reproductive function research.

**Main body of the abstract:**

This review provides a detailed description and discusses extensively the variations observed in different phases of the estrous cycle in laboratory animals using rats and mice as examples. It also illustrates how these phases can be determined and how to induce estrus ‘heat’ when required. The phases of the estrous cycle can be determined using various methods such as visual assessment, vaginal smear/cytology, histology of female reproductive organs (vagina, uterus and ovaries), vaginal wall impedance assessment and determination of urine biochemical parameters. Female animals can be artificially brought to estrus phase ‘heat’ to make them receptive to male counterparts.

**Conclusion:**

Determination of the length and phases of the estrous cycle and induction of estrus are useful in teaching and research and evaluating the effects of drugs/chemicals on the reproductive functions.

## Background

The importance of experimental animals in teaching and research cannot be over-emphasised. Studies using animal models have advantages such as the availability of experimental subjects, ability to perform invasive tests, extensive tissue sampling, and standardisation of disease severity and the possibility of prophylaxis [1]. Other advantages include secure handling of subjects, elimination of attrition from the study (except in cases of mortality), and controlled experimental conditions. Although, the choice of animal in biomedical research for a particular investigation may be controversial and highly opinionated, experimental animals commonly used include mice, rats, and rabbits. Small rodents like mice and rats are more preferred to larger animals like rabbits, dogs, cats, pigs and monkeys [2].

In reproductive function research, particularly those involving the use of female animals, mice and rats are commonly used, possibly due to their well characterized estrous cycle and secure handling. The short and precise length of estrous in these rodents [3, 4] also makes them very suitable. Determining the estrous phase is essential for selecting a female animal that will mate when paired with a male counterpart to achieve timed-pregnancy or tracking of estrous as a variable that may affect research [5]. Assessment of the estrous cycle in experimental animals is a useful measure of the integrity of the hypothalamic-pituitary-ovarian axis and the functioning reproductive status of the female reproductive system [4]. It can also be used to investigate the effects of drugs and chemicals on reproductive function [6] frequently expressed as a disruption in the typical morphology, cytology and histology of reproductive organs and alteration in the duration of particular phases of the estrous cycle.

### The estrous cycle

The estrous cycle refers to the reproductive cycle in rodents. It is similar to the human reproductive cycle, commonly called the menstrual cycle (ovarian and uterine cycles). The estrous cycle has four phases, namely proestrus, estrus, metestrus and diestrus and lasts for 4 to 5 days [4] (Table 1). The reproductive period and estrous cycle of mice commences about the 26th day after birth with the opening of the vagina, which is about 10 days before vaginal cornification [7]. The apoptosis-mediated vaginal opening is an essential secondary character in mice, which is used as a predictor of puberty [8]. The vaginal unfolding is associated with an increase in oestradiol concentration. In rats, a vaginal opening occurs during the first ovulation [4].

**Table 1:** Length of various phases of the oestrous cycle

Oestrous phase	Cycle length (hours)	
	Rats	Mice
Proestrus	14	< 24
Estrus	24–48	12–48
Metestrus	6–8	8–24
Diestrus	48–72	48–72

In female rats, puberty is preceded by the pulsatile release of luteinizing hormone (LH) after the 4th postnatal week, approximately 30 days old [9]. This period is the anestrus and occurs about 8 to 9 days before the first proestrus [10]. The first proestrus, estrus, metestrus and diestrus then follow. Metestrus only occurs in the absence of conception [6].

On the other hand, in humans, there are three phases of the menstrual cycle; the menstrual, proliferative (follicular), and secretory (luteal) phases. This cycle begins at puberty. On the average, it lasts about 28 days from the start of one menstrual period to the start of the next. At mid cycle, between the proliferative and secretory phases, is the ovulatory phase during which ovulation occurs following LH surge. The proliferative phase is primarily associated with high estrogen levels while the secretory phase is associated with high progesterone levels [11].

The proestrus phase corresponds to the human follicular stage, which is associated with a rise in circulating estradiol concentrations and little surge in prolactin, this leads to a rise in LH and Follicle Stimulating Hormone (FSH) release. The peak in FSH concentration with an associated rapid decline in estradiol levels correlates to ovulation and estrus phase. Metestrus and diestrus are homologous to human early and late secretory stages of the reproductive cycle, respectively, with high levels of progesterone [12].

### Historical perspective

The word “estrus” was first used by Heape [13]. This is a Latin adaptation of the Greek word “oistros” which means “sexual season”, “gadfly”, “frenzy”, “sting” or “madness” [14]. Heape further named and defined the phases of the mammalian estrous cycle into proestrus, metestrus, diestrus and anestrus. Proestrus is the preparatory stage for an animal coming into heat, metestrus which is a brief period characterized with decline of corpus luteum functions in the absence of conception when the activities of reproductive organs gradually subside, diestrus which is a period of short rest during the breeding season, and anestrus which a non-breeding period when reproductive organs are quiescent [15]. Behavioural changes, as well as morphological, cytological and histological changes in the reproductive tract, describe these phases.

In the early days, macroscopic changes in the vulva (such as vulva swelling), vaginal secretions (such as bleeding, and mucous), and uterus (such as congestion) as well as microscopic changes in the reproductive tract were used to define estrous cycle phases. However, macroscopic findings were unreliable in small rodents, and histological analysis was and remains an invasive procedure which is not suitable for estrous cycle staging in live animals. Stockard and Papanicolaou [16] later characterized the vagina changes during the estrous cycle through histology and cytology; this circumvented the earlier problems of unreliability and invasiveness. Over time, various studies have approved of Stockard and Papanicolaou’s assessment; this led to the existing definitions of the phases of the estrous cycle. The Pap smear by Papanicolaou is still used today as a screening for cervical cancer [17].

### Techniques for estrous cycle assessment

Several studies have proffered various methods of evaluating the estrous cycle based on the changes in the animal’s physiology and anatomy. These methods include visual assessment, [4, 5, 18] vaginal cytology, [3–5, 15, 19] histological examination of the reproductive organs, [6, 20] vaginal wall impedance, [21–23] and urine biochemistry [24].

### Visual assessment

The visual method of evaluating the estrous cycle is widely acceptable. It is non-invasive, simple, cheap, fast, less stressful to animals and researchers, and can be carried out anywhere and at any time provided the illumination is adequate. This method is as accurate and reliable as the vaginal smear [18]. It has been reported to be the fastest method to determine the estrus phase [5]. However, most of the studies that reported this method used mice; this could suggest that this technique is more suitable for small animals such as mice and rats. Findings from this technique are also observer-dependent.

In visual assessment of estrous phase, the mouse should be held in the non-dominant hand and laid in the restraint with the forepaws resting on a surface, lift the tail gently, then examine and evaluate the vulva based on the criteria of Champlin et al. [18] A digital image for documentation is essential. It is also important to avoid hasty examinations to prevent misinterpretations.

**Fig. 1:**
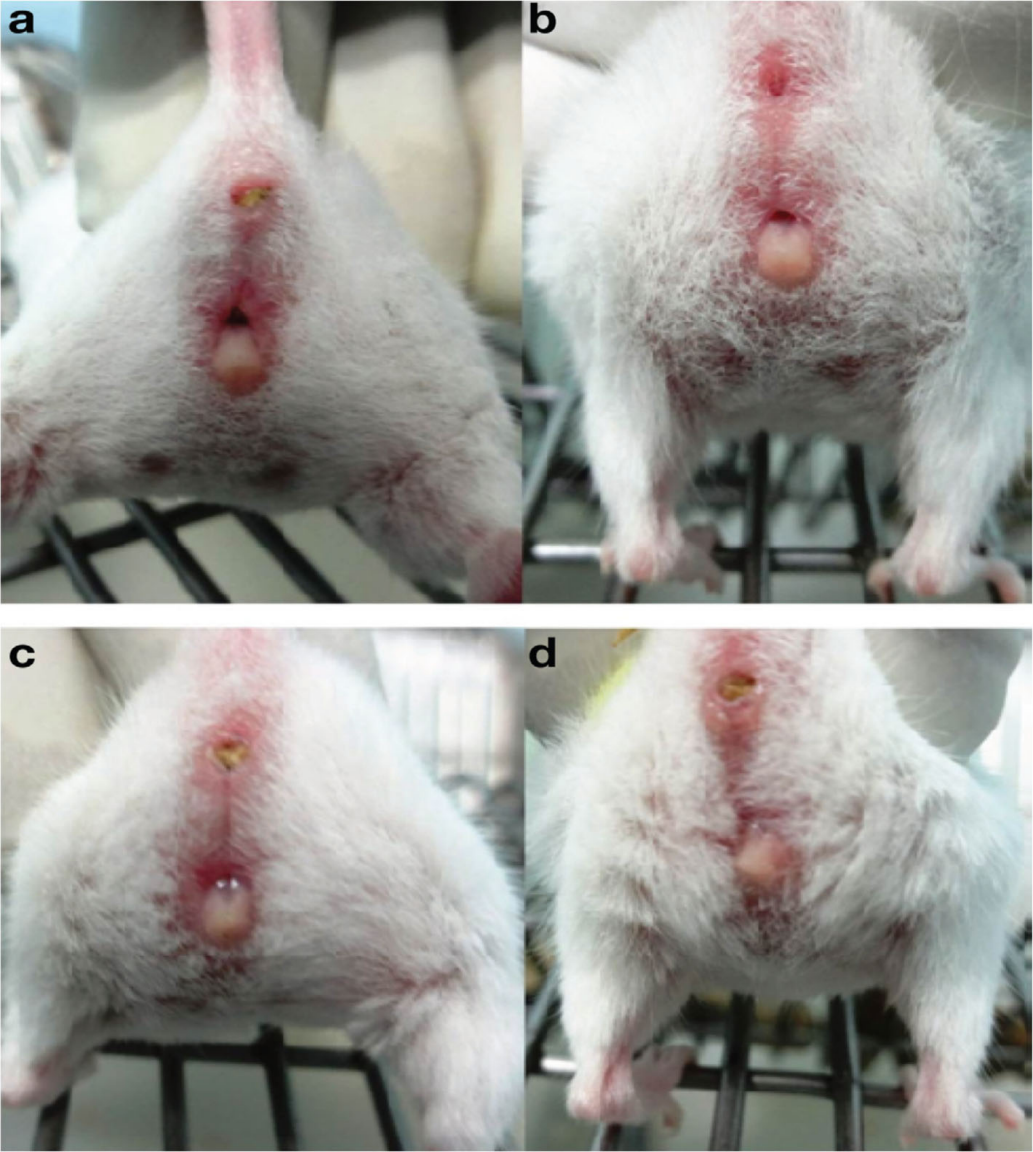
Appearance of the vagina in different phases of estrous cycle of a Swiss albino strain mouse. **a**-Proestrus, **b**-Estrus, **c**- Metestrus, **d**- Diestrus (Ekambaram et al., [9])

**Fig. 2:**
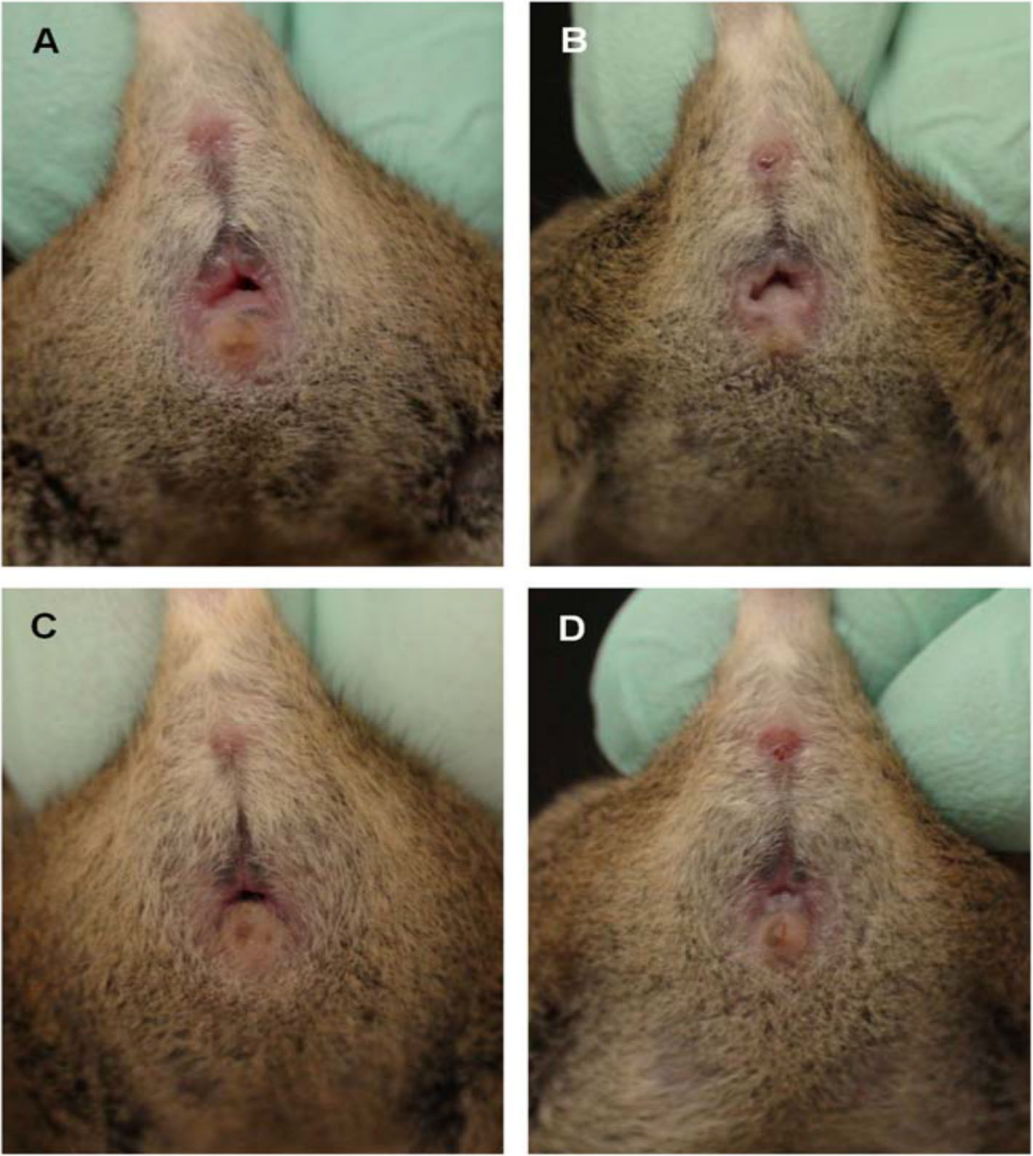
Appearance of the vagina in different phases of estrous cycle of an agouti strain mouse. **a**-Proestrus, **b**-Estrus, **c**- Metestrus, **d**- Diestrus (Byers et al., [6])

**Fig. 3:**
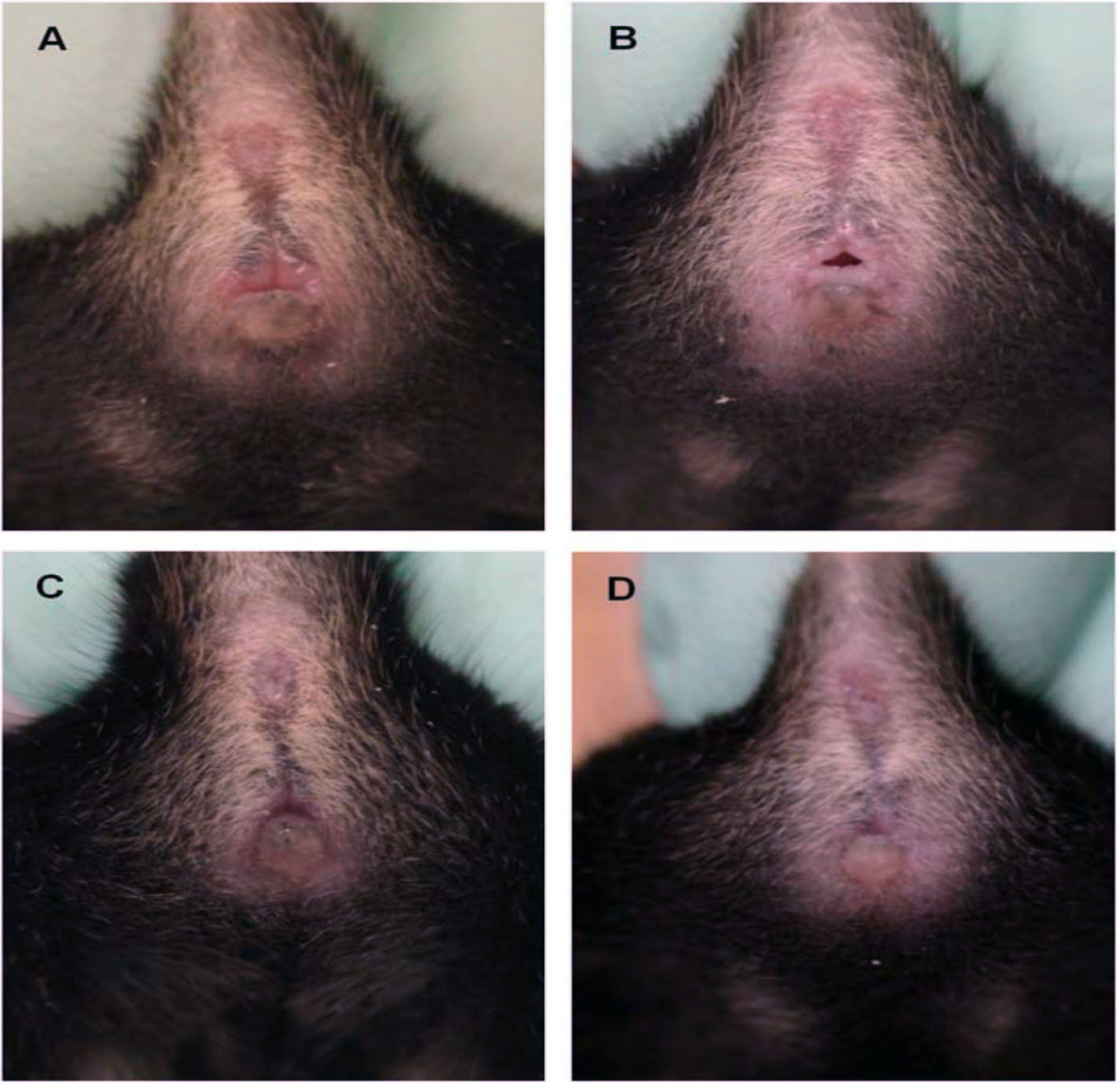
Appearance of the vagina in different phases of estrous cycle of a non-agouti strain mouse. **a**-Proestrus, **b**-Estrus, **c**- Metestrus, **d**- Diestrus (Byers et al., [6])

The appearance of the vagina at various phases of the oestrous cycle is summarized in Table 2. In the proestrus phase, the vaginal opening appears full, swollen and moist. The tissues are pink, with striations in both the dorsal and ventral lips of the vulva. In estrus, the vagina appears similar to that in the proestrus, but it is less pink, less swollen and less moist with more prominent striations. In metestrus, the vagina opening is pale and dry with a slough of white cellular debris. The features of diestrus include a very wet vaginal opening, which is sometimes too small and closed in some mice with no tissue swelling [4, 5] (Figs. 1, 2 and 3).

**Table 2:** Appearance of the vagina at different phases of the oestrous cycle (Champlin et al., [7])

Oestrous Phase	Appearance
Proestrus	Vagina is gaping and the tissues are moist and reddish-pink. There are numerous longitudinal folds or striations visible on the dorsal and ventral lips.
Estrus	Vaginal appears similar to that seen at proestrus, but the tissues are lighter pink and less moist. The striations are more prominent.
Metestrus 1	Vagina tissues are pale and dry. The dorsal lip is not as oedematous as in the estrus
Metestrus 2	Vagina appears similar to that seen at Metestrus 1, but the lip is less oedematous and has receded. Whitish cellular debris may line the inner walls or partially fill the vagina.
Diestrus	Vagina is moist and has a small opening and the tissues are bluish-purple in colour.

### Vaginal smear/cytology

Similar to the visual assessment, vaginal cytology is also widely accepted. It seems to be the most common technique used to determine the phases of the estrous cycle. It is non-invasive and relatively inexpensive. Although this method requires some measure of skill for microscopic examination of the vaginal secretion cells, it is accurate and reliable. However, this method has also been reported to be tedious and time-consuming [22].

During assessment, the animal and its forepaws are restrained. The tail is elevated to visualize the vagina. The vaginal cells are flushed by gently introducing a little amount (100 μl) of distilled water or saline using a pipette or sterile latex bulb. Phosphate-buffered saline can be an alternative fluid. Slowly release the liquid into the vaginal and draw it back into the tip; this should be repeated about 4 to 5 times in the same sterile latex bulb. It is essential to ensure that the pipette or sterile latex bulb is placed at the entrance of the vaginal canal and does not penetrate the vaginal orifice. The fluid containing few drops of cell suspension is after that introduced on a glass slide, air-dried and stained accordingly. 0.1% crystal violet stain (0.1 g of crystal violet powder in 100 ml of double distilled water) may be used [4]. Alternative stains are Romanowsky-type stains (such as Modified Wright’s, Wright’s Giemsa) and Toluidine blue O [15]. The slide should then be overlaid with a coverslip and examined under a light microscope immediately at 200 X magnification.

Alternatively, a vaginal swab can be obtained using cotton-tipped swab wetted with ambient temperature physiological saline and introduced into the vagina of the restrained mouse [5]. The swab should be gently turned and rolled against the vaginal wall and then removed. Cells collected are then transferred to a dry glass slide by moving the swab across the slide. The slide is air-dried, stained, and viewed under the microscope [5].

**Fig. 4:**
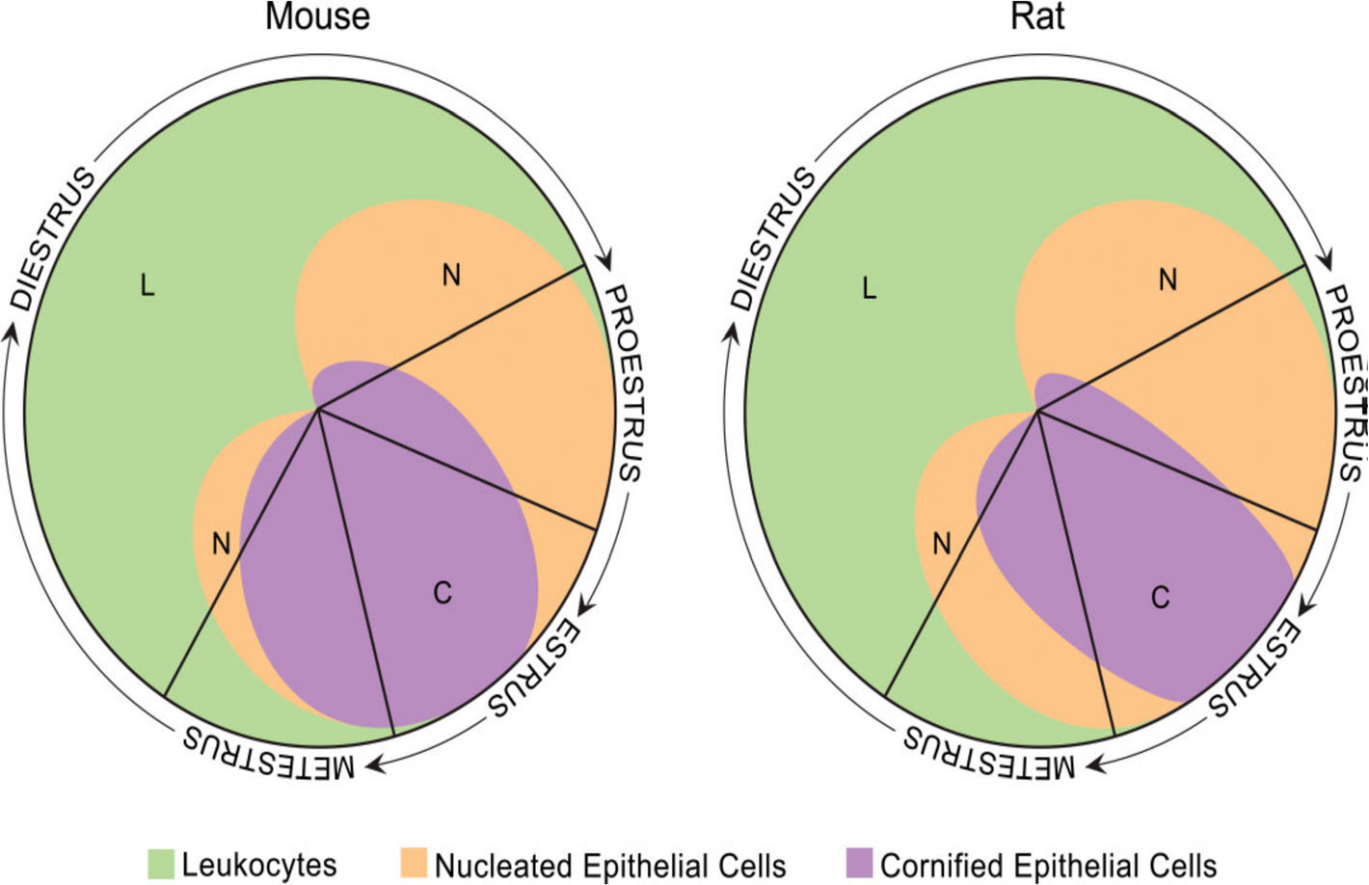
Oestrous cycle wheel

The vaginal secretion is made up of three types of cells. They include leucocytes, cornified epithelial cells and nucleated epithelial cells. Estimation of the phase of estrous cycle is based on the proportion of these cells in the vaginal secretion [4] (Table 3 and Fig. 4).

**Table 3:** Classification of the phases of oestrous cycle based on the cell types and relative number of these cells in vaginal smears (Cora et al., [8])

Oestrous phase	Neutrophils	Small nucleated epithelial cells	Large nucleated epithelial cells	Anucleated keratinized epithelial cells	Relative cell density
Proestrus	0 to +	++ to +++	0 to +	0 to +	Low to moderate
Estrus					
Rat	0 to +	0 to ++	0 to ++	++ to +++	Moderate to high
Mouse	0 to +	0 to +	0 to +	++ to +++	Moderate to high
Metestrus					
Rat	+ to +++	+ to ++	+ to ++	+ to +++	Moderate to high
Mouse	+ to +++	0 to +	0 to +	++ to +++	Moderate to high
Diestrus	++ to +++	+ to ++	+ to ++	0 to +	Low to moderate

0 = none; + = few; ++ = moderate; +++ = high

**Fig. 5:**
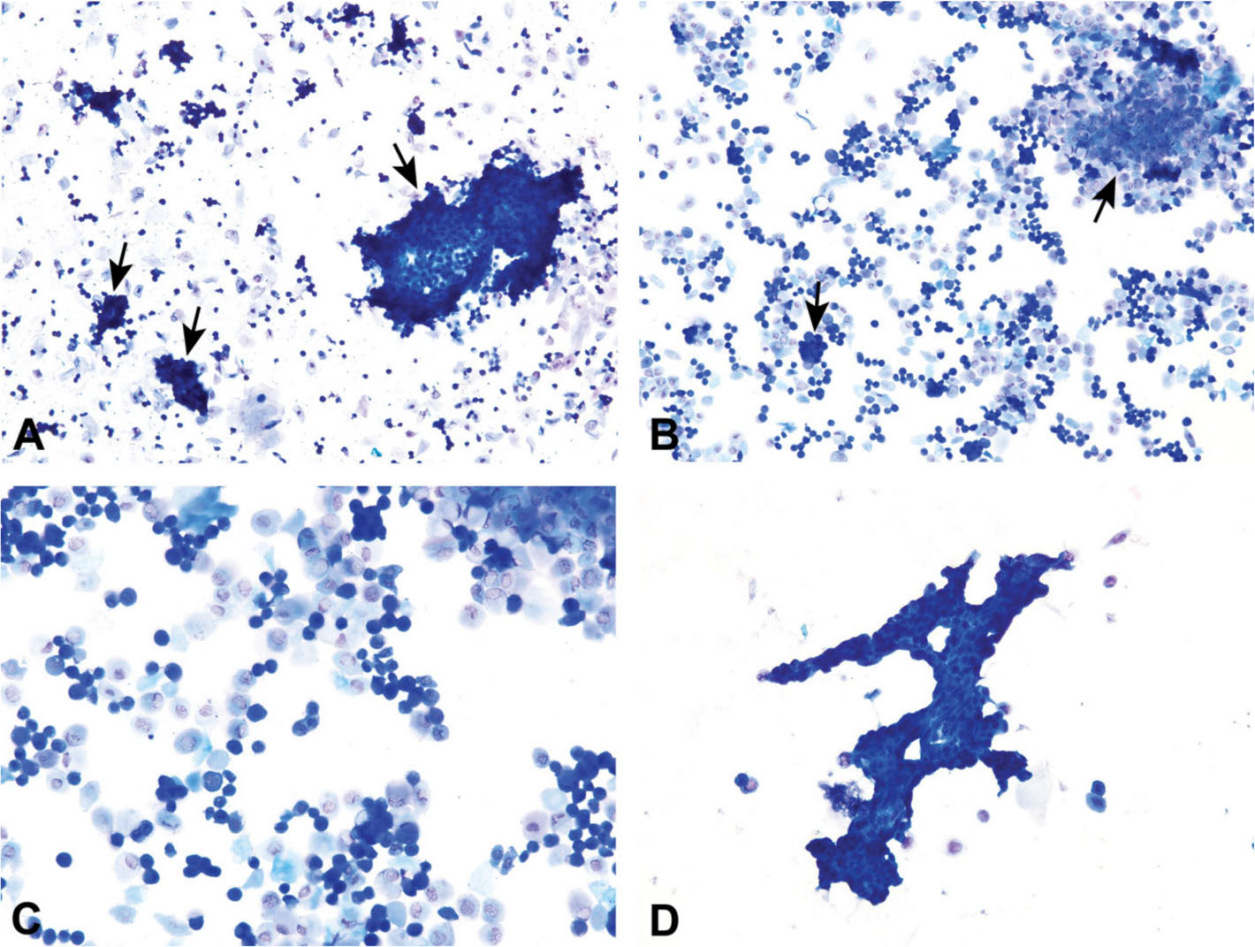
Vaginal smears at proestrus in Sprague-Dawley rats

**Fig. 6:**
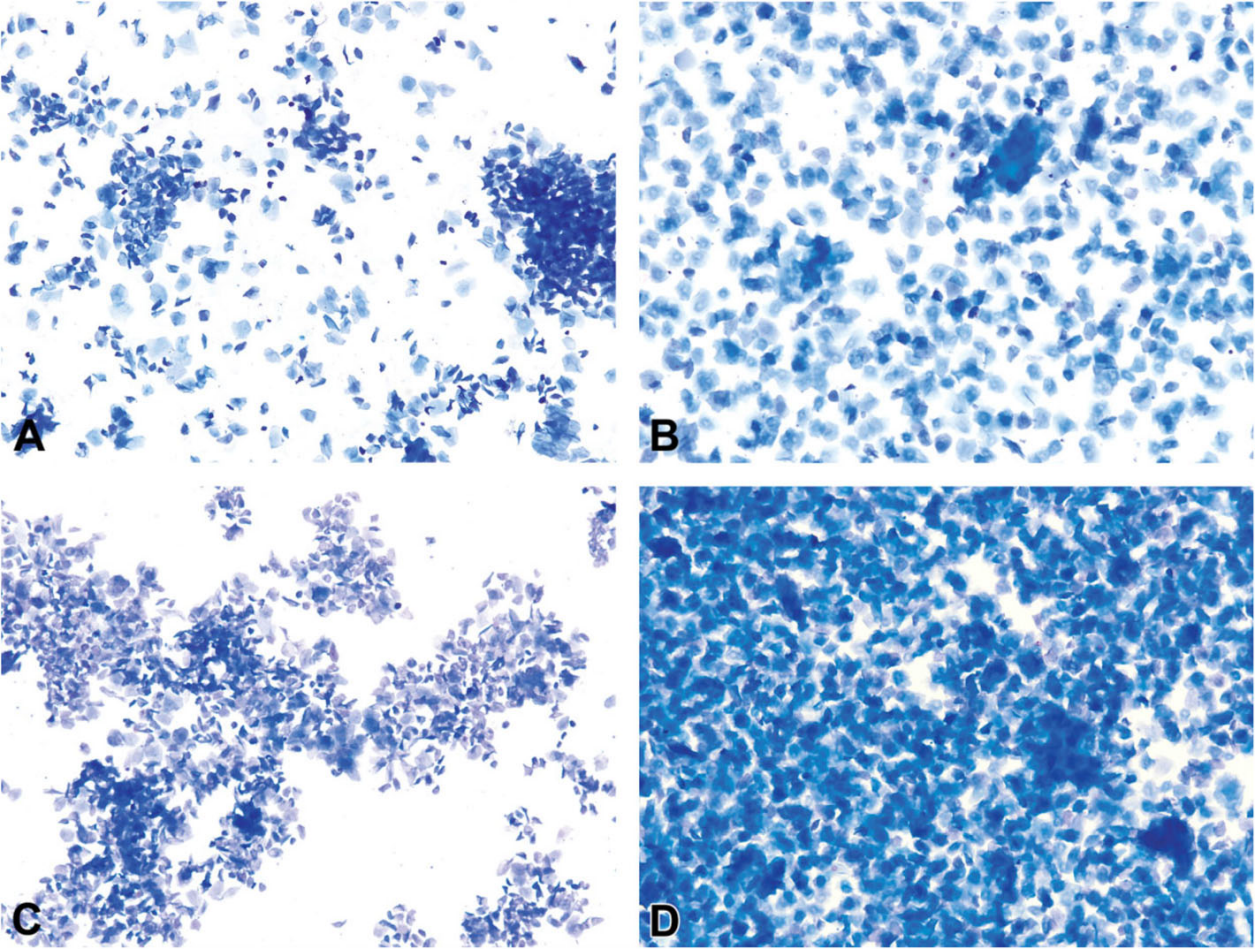
Vaginal smears at estrus in mice

**Fig. 7:**
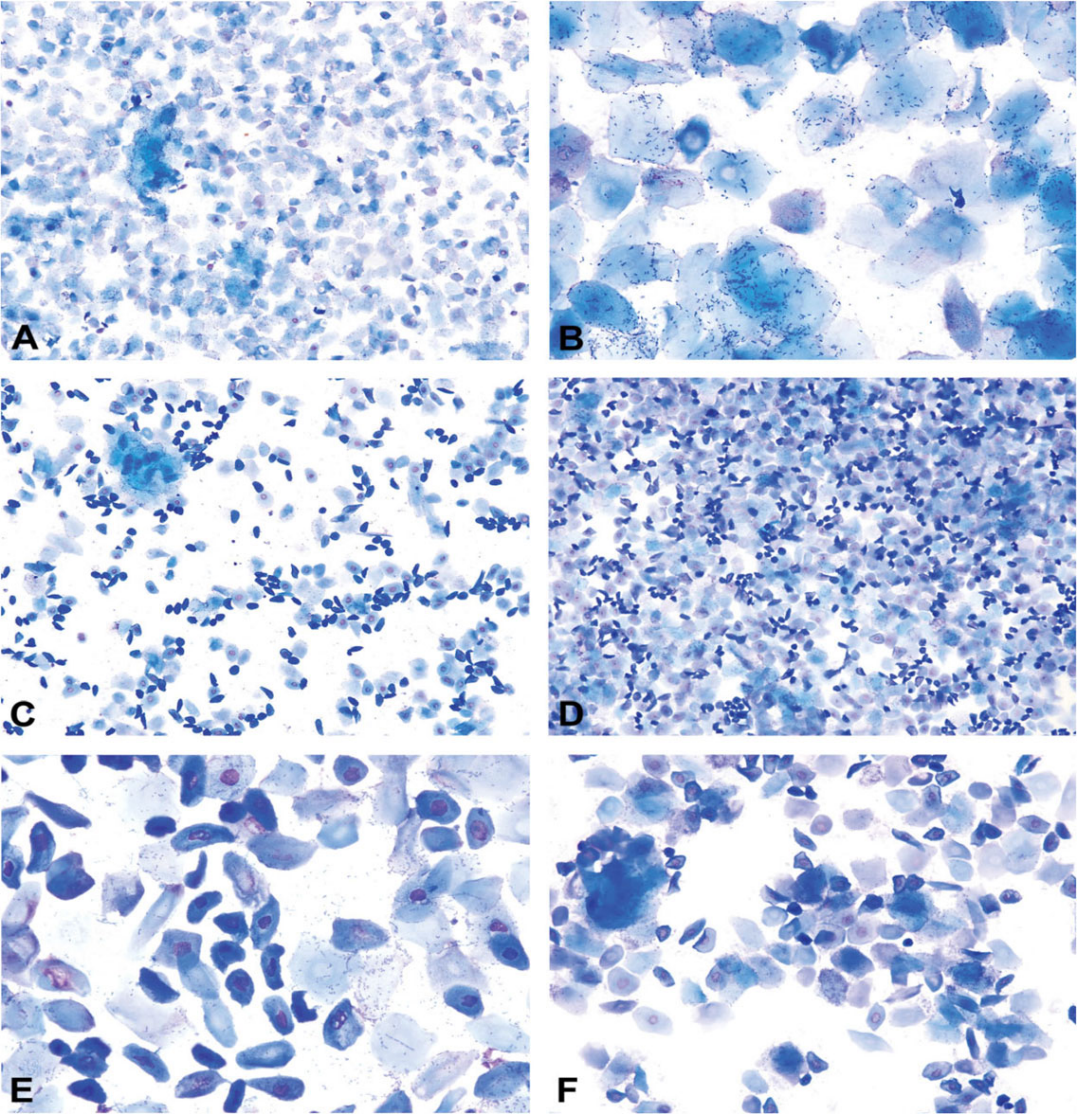
Vaginal smears at estrus in Sprague Dawley rats

**Fig. 8:**
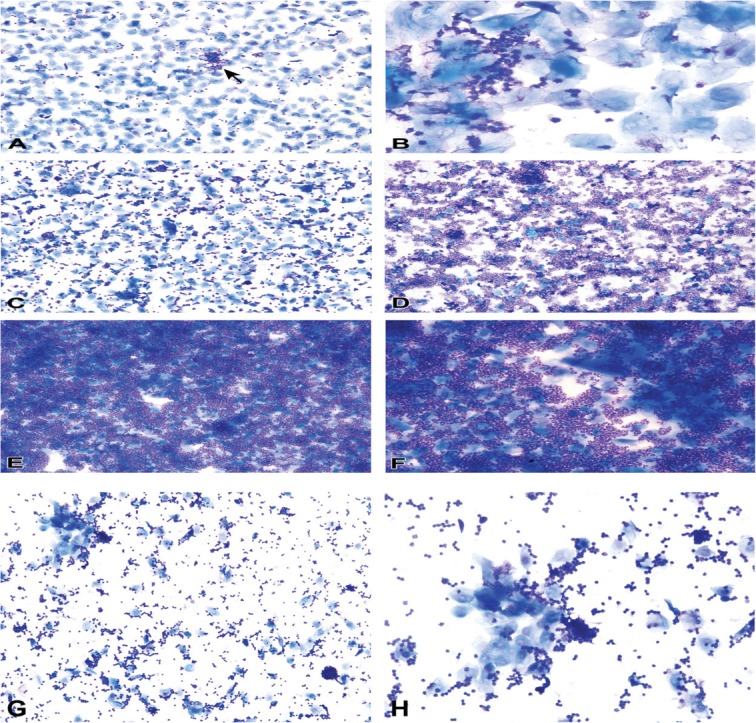
Vaginal smears at metestrus in mice

**Fig. 9:**
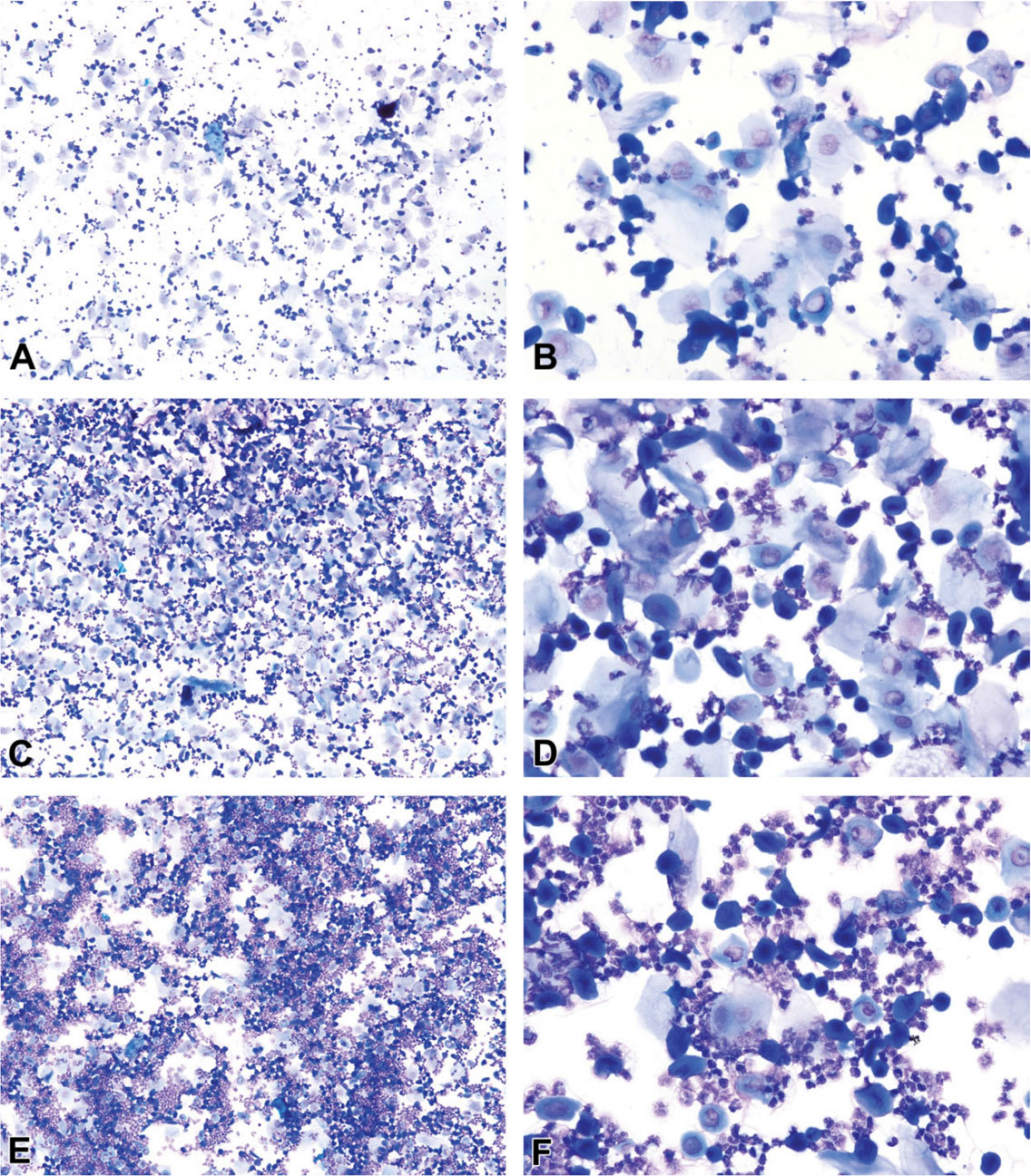
Vaginal smears at metestrus in Sprague Dawley rats

**Fig. 10:**
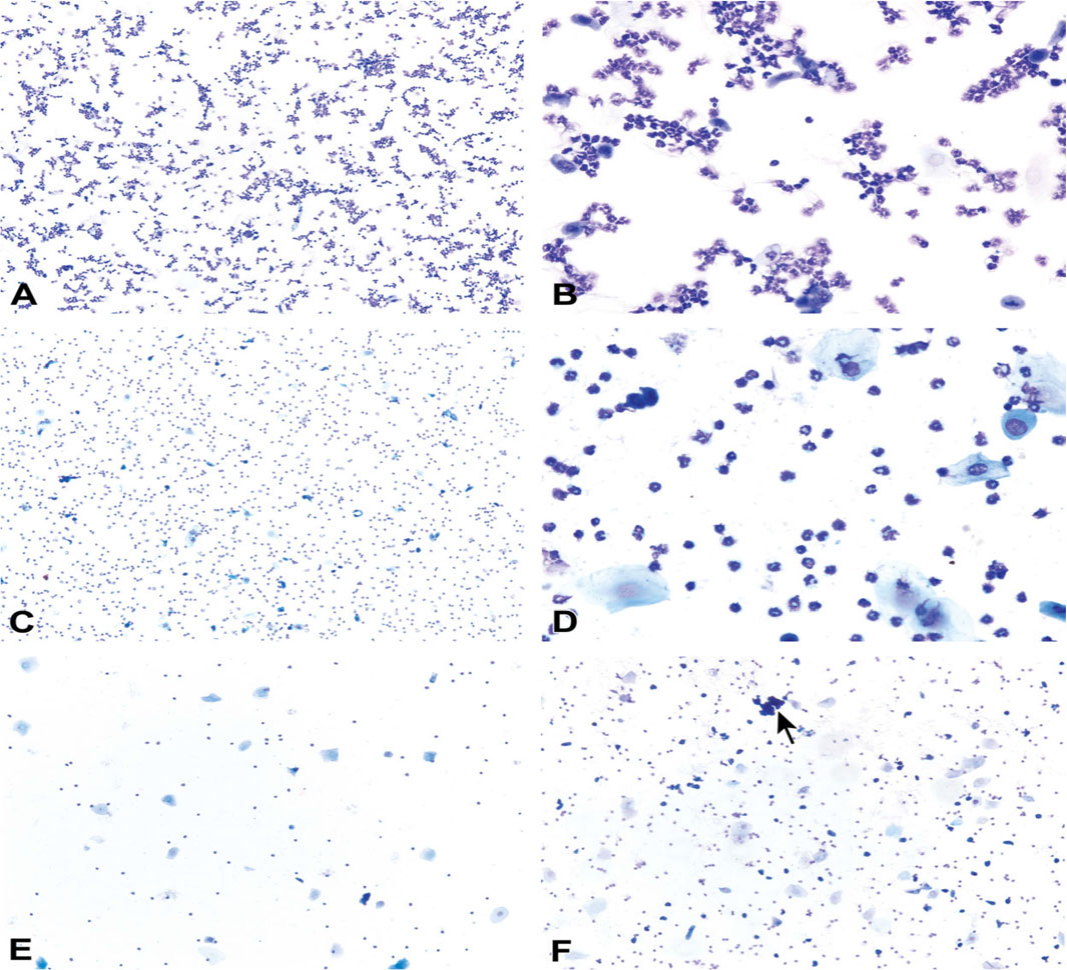
Vaginal smears at diestrus in mice (**a**-**e**) and Sprague Dawley rats (**f**)

Numerous round nucleated cells which are uniform in size and appearance characterize the proestrus phase. They appear in clusters or individually. There are also few anucleated cornified epithelial cells. Some white blood cells may be present in the female in early proestrus (Fig. 5). The estrus phase shows abundant anucleated cornified epithelial cells. The cytoplasm is granular, and the cells are irregular in shape. Also observed are numerous bacteria and occasionally, nucleated epithelial cells (Figs. 6 and 7). At metestrus, a large number of leucocytes and a small number of large, non-granular and anucleated cornified epithelial cells are seen. The formation of corpus luteum which fails to fully luteinize due to lack of progesterone and results in sloughing off of the uterine lining accounts for the cornified epithelial cells and polymorphonuclear leukocytes present in vaginal swabs. Some nucleated epithelial cells may also be present in late metestrus (Figs. 8 and 9). Diestrus shows prominent polymorphonuclear leukocytes and a few epithelial and cornified cells. Leukocytes remain the predominant cell type having removed cellular debris [4, 5, 15] (Fig. 10).

### Histological examination of the reproductive organs

Although the determination of the phases of the estrous cycle by this technique is very reliable and accurate, it comes with many drawbacks. This technique is invasive and not useful for estrous assessment in life laboratory animals. It is also costly and requires a high measure of skill. Also, it is too tedious and time-consuming. This method is not readily available, hence not feasible particularly in remote areas of the tropical region.

The minimal requirement for estrous cycle evaluation by histology is the complete longitudinal sections of the vagina and cervix, transverse sections of the mid-portion of both uterine horns, and middle sections of both ovaries [6]. The presence of ovarian follicles cannot be used to determine the phase of estrous in rats and mice with short estrous cycles. Follicles are almost always present in the ovaries at all stages. However, the appearance of particular morphologies of corpus luteum can help in staging even though it may not be definitive [25]. The excised organs are fixed with 10% formalin, stained with hematoxylin and eosin, and examined microscopically.

**Fig. 11:**
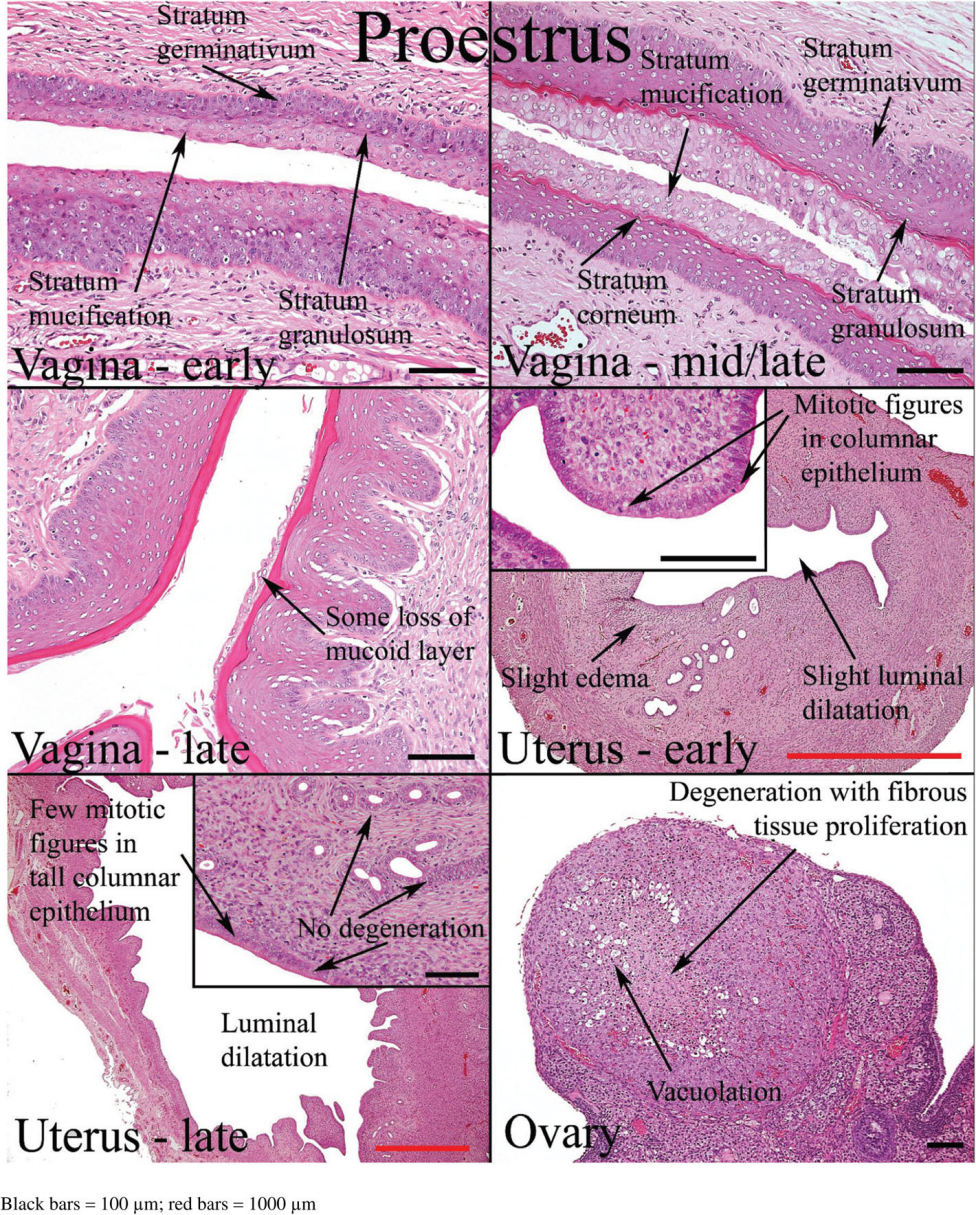
Histology of the rat reproductive organs at proestrus (West wood, 2008)

**Fig. 12:**
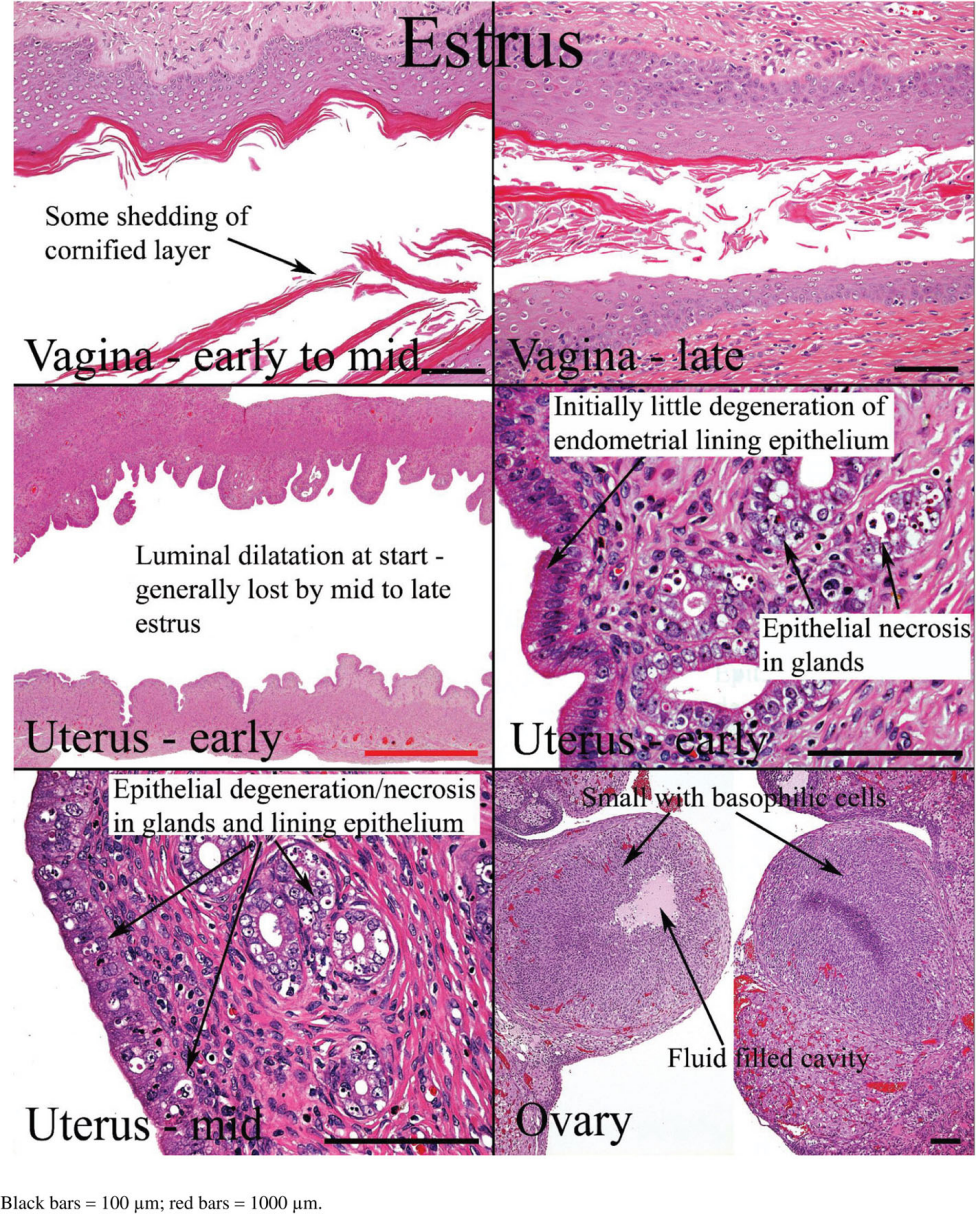
Histology of rat reproductive organs at estrus (West wood, 2008)

**Fig. 13:**
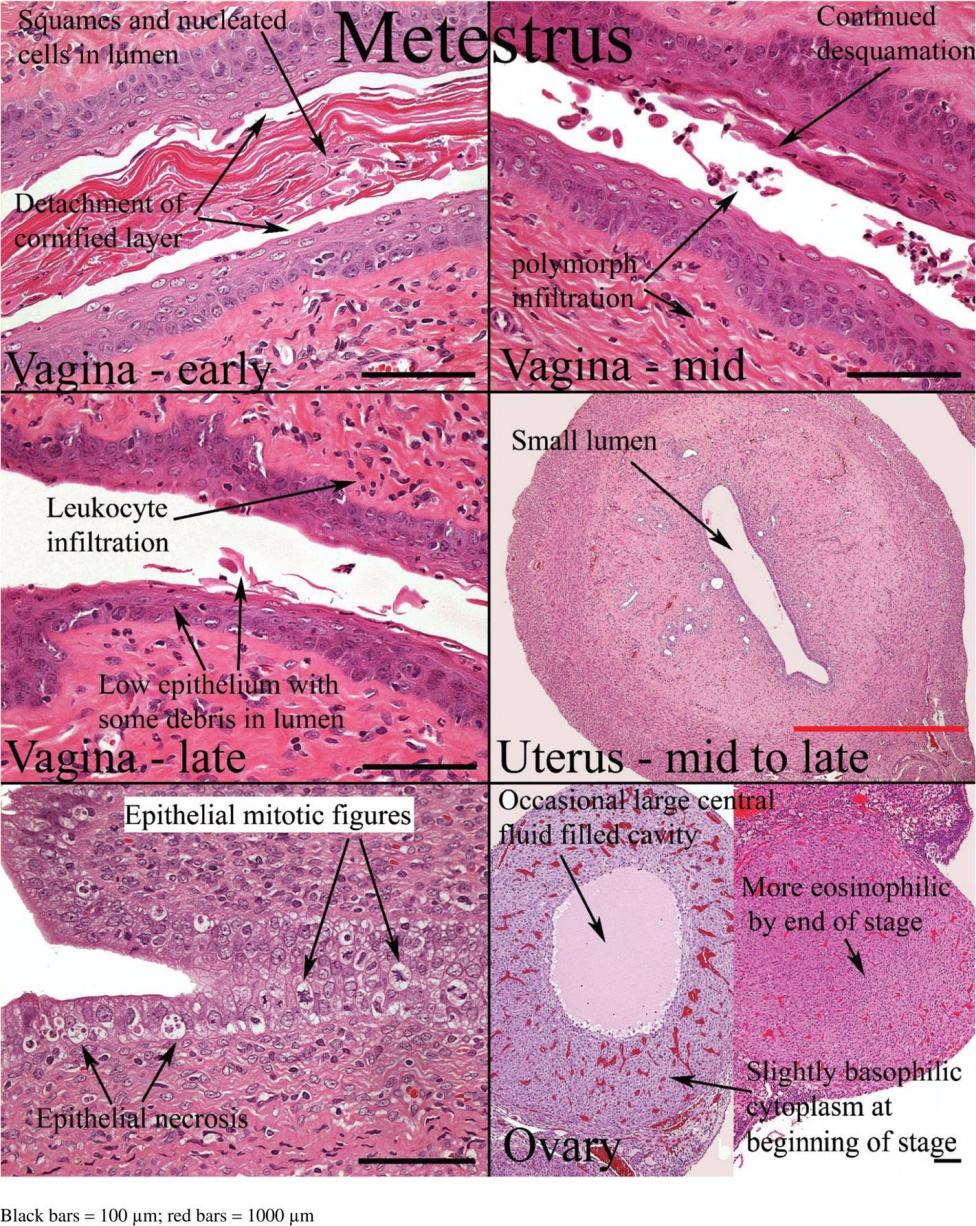
Histology of the rat reproductive organs at Metestrus (Westwood, 2008)

**Fig. 14:**
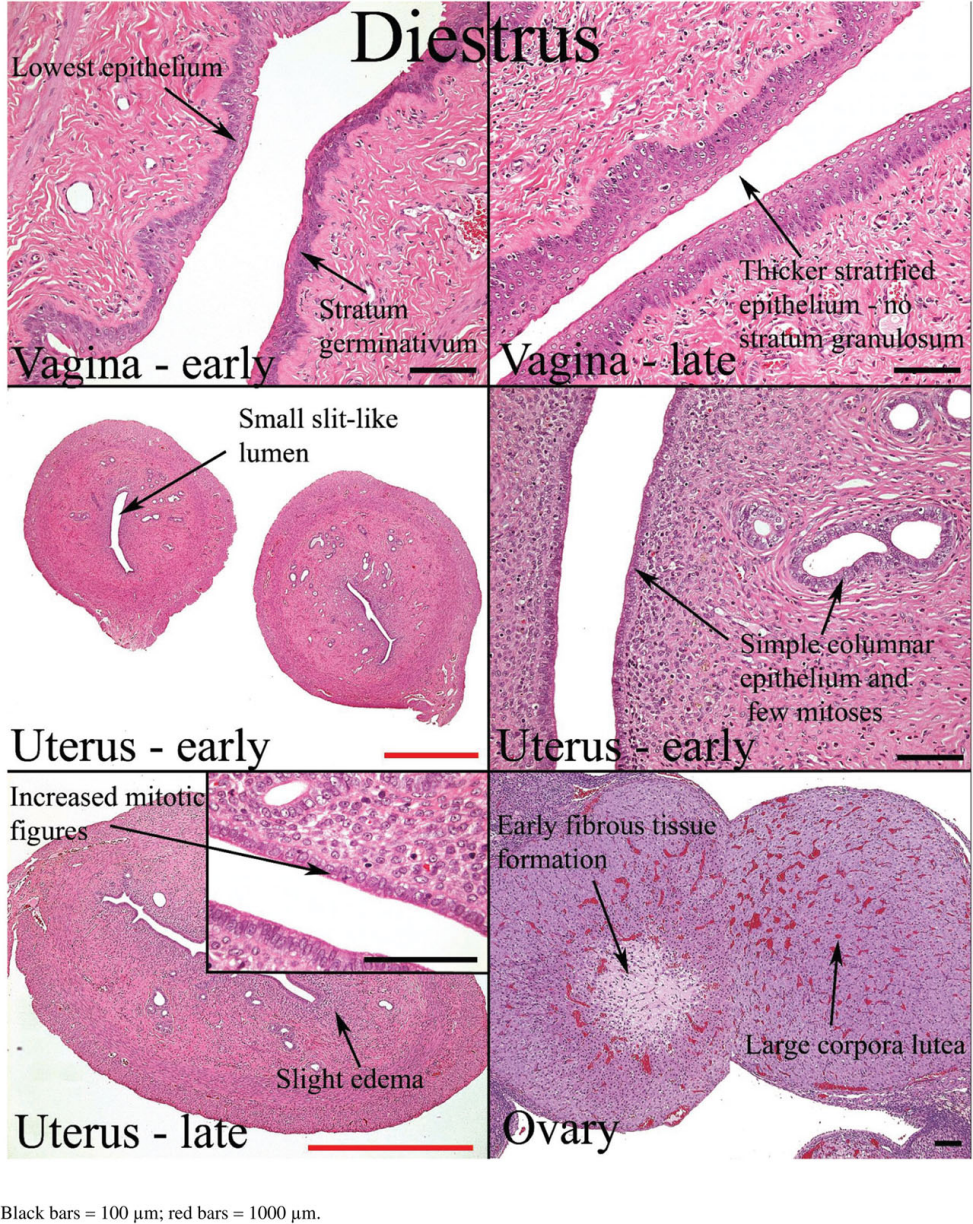
Histology of the rat reproductive organs at diestrus (Westwood, 2008)

Histological appearances of the reproductive organs at various estrous phases are shown in Table 4 and Figs. 11, 12, 13 and 14.

**Table 4:** Histological features of the rat reproductive tract during different phases of oestrous cycle (Westwood, 2008)

Oestrous phase	Vagina	Uterus	Ovaries
Proestrus	Presence of mitotic figures. Polymorphs are occasionally present. There is little or no degeneration or desquamation. Formation of stratum granulosum (indicates onset), superficial mucoid layer and corneum with time. At the end of this phase, cornified cells are prominent with superficial mucoid layer and some desquamation mucoid cells.	Epithelium is cuboidal to columnar. There is presence of mitoses in epithelial cells with little or no degeneration and little infiltration of inflammatory cell. Dilatation is seen towards the end of proestrus	Corpus luteum degenerates. There is the presence of cytoplasmic vacuoles and fibrous tissue proliferation in the central cavity
Estrus	There is gradual shedding of superficial mucoid and cornified layers with reduction in height of epithelium. There is appearance of cell debris with loss of mitotic figures and gradual leukocyte infiltration.	The onset is characterized by appearance of notable degeneration/necrosis of epithelial cells. There is loss of mitotic activity and leukocyte infiltration. Dilatation may persist to late estrus.	Degenerated corpus luteum is usually present. There are small corpus luteum with basophilic cell cytoplasm, central fluid-filled cavity and no fibrous tissue.
Metestrus	At the onset, there is complete detachment of cornified layer. Leukocyte infiltration persists with continued desquamation and loss of stratum granulosum and upper germinativum.	There is continued degeneration of endometrial epithelial cells. Mitotic activity is seen; both mitotic activity and degeneration are seen together.	Corpus luteum may still contain fluid cavity. There is minimal basophilic cells. No fibrous tissue.
Diestrus	The onset is characterized by epithelium with variable leukocyte infiltration, and subsequent epithelial proliferation and thickening. There is no leukocyte infiltration at the later part of this phase.	There is a small, avascular, slit-like lumen. There is low columnar epithelium with few mitoses which increases as the cycle progresses. Stromal oedema is seen at the end of this phase.	Large corpus luteum is seen. There is fibrous tissue formation in the central cavity.

### Vaginal wall impedance

Although the use of electrical impedance on the vaginal wall to determine the phase of estrous is controversial and is rarely cited in the literature [22], it is seemingly gaining more attention. This method is convenient, readily available, and requires less skill. It is much less amenable to subjective interpretation on the part of the technical operator than the more conventional vaginal smear methodology [22]. However, it is also expensive and not widely accepted, possibly due to its unreliability. This technique rather differentiates estrus from non-estrus animals.

**Fig. 15:**
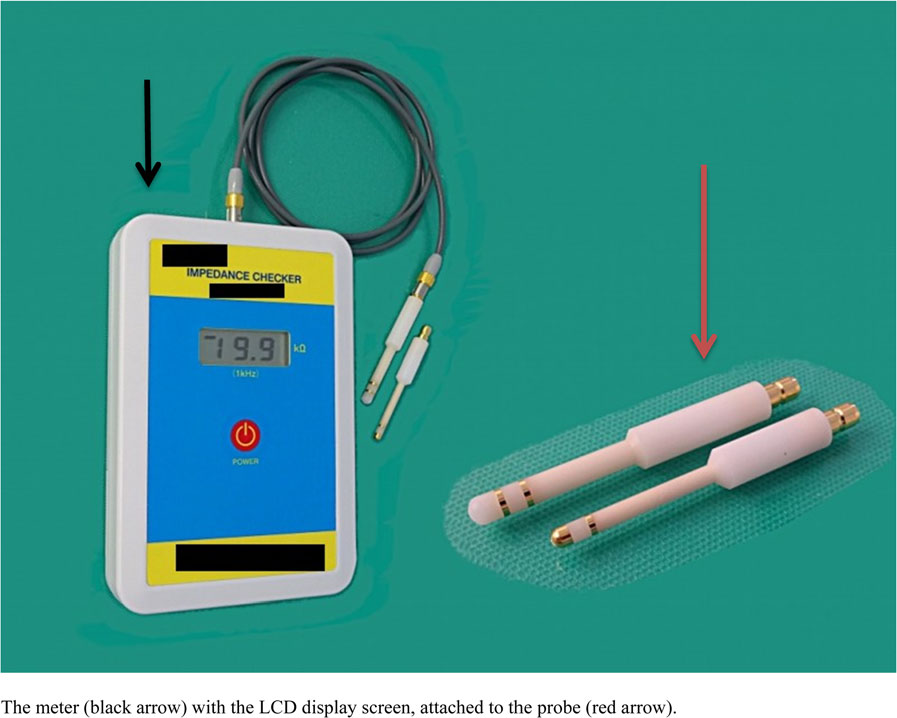
A standard impedance monitor

**Fig. 16:**
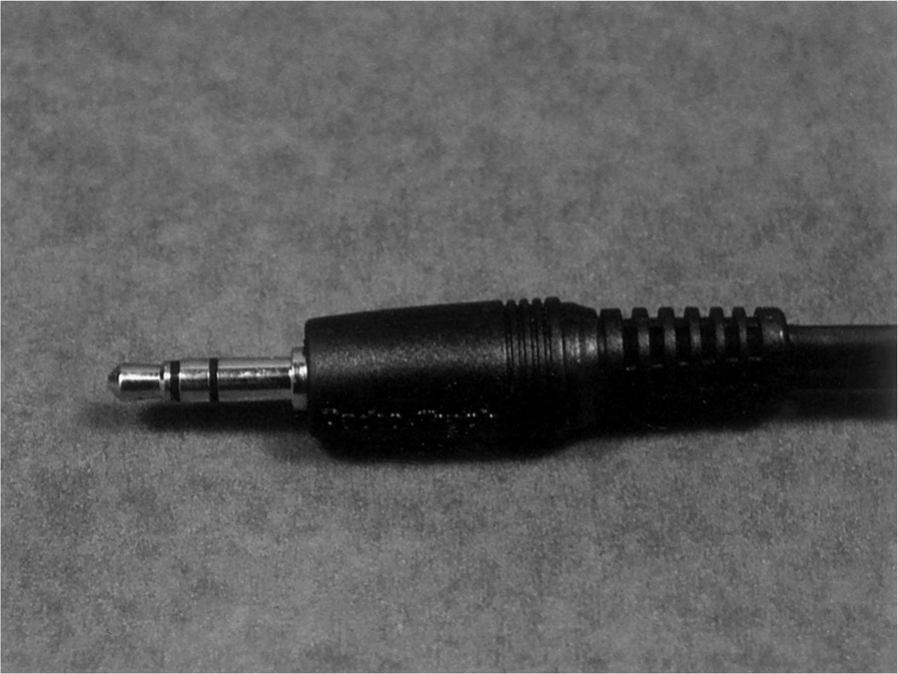
Male terminal of a standard stereo audiocable used as a resistance-measuring probe (Ramos et al., [20])

An impedance monitor used for this technique is made up of a meter and vaginal probe (Fig. 15). One person restrains the animal while another person inserts the vaginal probe for about 30 s to take impedance measurements [23] on the display screen of the meter. The probe is made clean with 70% alcohol before each analysis. Alternatively, a simple, cheap, and portable battery-operated electrical meter that measures resistance over a wide range, which is commonly used by electricians can be employed as described by Ramos et al. [22] When using this alternative device, leads fabricated from the internal wiring of a typical inexpensive shielded audio cable should be attached to it, and a male terminal used as a vaginal probe (Fig. 16).

Animals in estrus have higher vaginal impedance than those in non-estrus phase. Jaramillo et al. [21] revealed that pairing of a female with male animals based on vaginal wall impedance resulted in higher rates of confirmed mating as well as pregnancy when compared to pairing based on the vaginal smear. On the other hand, Singletary et al. [23] observed that rats in estrus phase as determined by vaginal cytology had higher vaginal impedance than those in non-estrus, but vaginal impedance and estrous cycle phase as determined by vaginal cytology did not correlate. Most authors who documented the use of vaginal wall impedance verified their findings with vaginal cytology; hence, no benchmark value has been reported to indicate whether or not an animal is in estrus.

### Urine biochemistry

Although this method of estrous assessment is yet to gain popularity and universal acceptance, it is a useful tool. This technique is simple, relatively cheap and not tedious. Since the levels of urine carbohydrates, proteins and fatty acids are usually in response to oestradiol and progesterone; this could vary across species and strains. This method is non-specific and unreliable because some animals have consistently high protein levels in their urine, which serve as chemical signals in these animals [24].

During assessment, the experimental animal should be held over a watch glass, and a gentle abdominal massage applied to stimulate micturition. The urine sample is collected and screened through a cheesecloth or nylon mesh (16-120 μm) at the time of collection. Sample should be stored at − 20 °C if not analysed immediately [24]. Urine carbohydrates, proteins, and lipids/fatty acids should be analysed using standard methods.

Urine protein and lipid levels are significantly higher in proestrus and estrus phases. The concentration of fatty acids in the urine is also very higher in estrus. Urine carbohydrate level is similar throughout the estrous cycle [24]. Like the vaginal wall impedance, there is no report of standard values for urine biochemical at each phase of the estrous cycle for commonly used laboratory animals.

### Induction of estrus

When assessing sexual behaviour, particularly in male animals, or the aphrodisiac potentials of a particular agent/drug, it is essential to bring the female animals to artificial estrus (phase)/heat. This increases their receptivity and readiness to mate the male counterparts. This practice is common in animal husbandry. It is achieved by administering suspension of ethinylestradiol at 100 μg/animal per oral 48 h before pairing with male counterpart and progesterone at 1 mg/animal subcutaneously 6 h before matching [26]. Alternatively, estradiol benzoate at 10 μg/100 g body weight and progesterone at 0.5 mg/100 g body weight are administered subcutaneously 48 h and 4–6 h respectively before pairing [27–29] (Table 5). Estrus phase is confirmed commonly by vaginal smear [30]. A combination of visual assessment with vaginal smear can establish estrous. Confirmation of the estrous phase of the induced female animal is by the assessment of its receptivity. This is done by exposing them to a male counterpart (not used for the study) prior to the experiment/test. Female animals with maximum receptivity are allowed to mate.

**Table 5:** Induction of estrus phase (heat)

Method	Procedure	Reference(s)
Common method	Subcutaneous Oestradiol benzoate 10 μg/100 g body weight 48 h prior to pairing + subcutaneous progesterone 0.5 mg/100 g body weight 4–6 h prior to pairing	(Amin et al., [2]; Sahoo et al., [22]; Tang et al., [26])
Alternative method	Oral ethinyl oestradiol 100 μg/animal 48 h prior to pairing + subcutaneous progesterone 1 mg/animal 6 h prior to pairing	(Singh et al., [23])

## Conclusions

Summing up, it is worth to know that various techniques can determine the phases of estrous cycles. Careful selection of the method to employ is essential. Vaginal smear/cytology remains the gold standard in life animals upon which other methods are verified. Although histological examinations of reproductive organs are as specific and reliable as vaginal smear/cytology, it is not useful in live animals. Improvement of the available techniques to enhance their reliability and specificity is pertinent. Studies to achieve benchmark values for vaginal wall impedance and urine biochemical parameters at different phases of estrous in commonly used experimental animals is also essential.

## Data Availability

Not applicable.
